# Sociodemographic determinants of multimorbidity in asthma: Insights from a retrospective cross-sectional study of the Obstructive Lung Disease program, Pakistan

**DOI:** 10.12669/pjms.42.(ICON26).15698

**Published:** 2026-04

**Authors:** Madiha Siddiqui, Sheikh Hina Sadiq, Shumaila Zeeshan, Saima Saeed

**Affiliations:** 1 Madiha Siddiqui (FCPS Pulmonology), The Indus Hospital & Health Network Indus Hospital & Health Network, Korangi, Karachi, Pakistan; 2 Sheikh Hina Sadiq (MSc Genetics), The Indus Hospital & Health Network Indus Hospital & Health Network, Korangi, Karachi, Pakistan; 3 Shumaila Zeeshan (MCPS Family Medicine), The Indus Hospital & Health Network Indus Hospital & Health Network, Korangi, Karachi, Pakistan; 4 Saima Saeed (FRCP, London), The Indus Hospital & Health Network Indus Hospital & Health Network, Korangi, Karachi, Pakistan

**Keywords:** Asthma, Multimorbidity, Obstructive Lung Disease, Sociodemographic determinants

## Abstract

**Background & Objective:**

Multimorbidity (MM) or the presence of additional chronic conditions in asthma, worsens disease control and increases healthcare use, but its prevalence and sociodemographic links in Pakistan are unclear. Our objectives were to assess the prevalence and patterns of MM in asthma and examine associations with key social and demographic factors.

**Methodology:**

This observational cross sectional retrospective study was carried out at nine primary care sites of Indus Hospital & Health Network (IHHN), Pakistan. Obstructive Lung Disease (OLD) program data from January 2021 to March, 2025 of 5,802 physician confirmed asthma patients aged 12 years and above were analyzed using Statistical Package for Social Sciences (SPSS). Frequencies, percentages and logistic regression assessed prevalence, patterns, and associations of MM with age, gender, location, employment in asthma.

**Results:**

Amongst 5,802 patients, 19% had MM, most commonly hypertension and diabetes mellitus. Prevalence was highest in middle-aged (22.5%) and older adults (31.2%), while 6.2% of adolescents and young adults were also affected. After adjusting for age, gender, geographical location, and employment status, the odds of having MM were significantly higher among middle-aged and older adults compared with adolescents and young adults (p < 0.001). Male sex, rural residence, and non-employment were also associated with MM (p < 0.05). Having >1 comorbidity was more likely in older (AOR = 15.72) and middle-aged (AOR = 9.63) adults than in younger groups, and in females versus males (AOR = 1.85; all p < 0.001).

**Conclusion:**

Asthma patients in Pakistan face a substantial MM burden influenced by sociodemographic factors, highlighting the need for patient-centered care.

## INTRODUCTION

Asthma, a major Chronic Respiratory Disease (CRD), substantially impacts the health and quality of life of individuals across the globe. In 2021, the Global Burden of Diseases study reported 39 million prevalent cases worldwide, with the highest recorded asthma deaths of 232.82 thousand in the South Asia region, including Pakistan. This was associated with the lower Socio-Demographic Index in this region, a composite score to evaluate the overall status of social and economic development of a country.^1^ Another major contributor to fatal exacerbations in asthma is multimorbidity, defined as the coexistence of two or more chronic conditions within the same individual. This is increasingly recognized as a major challenge in the management of all non-communicable diseases.^2^

Multimorbidity (MM) in asthma is linked to common pathophysiological mechanisms between asthma and other chronic conditions, central to which is chronic systemic inflammation. This may be triggered by immune dysregulation, metabolic effects of treatments like corticosteroids, neuroendocrinal stress responses, and gut–lung axis alterations.^3^ Additionally, aging and environmental factors like smoking, air pollution, physical inactivity, and poverty have been known commonly identified in patients diagnosed with asthma, as well as other non-communicable conditions.^4^

Holistic care is required to recognize asthma as part of a multimorbid state. The Global Initiative for Asthma emphasizes finding and managing coexisting conditions due to association with poor health and increased healthcare burden.^5^ In a meta-analysis, over 5.4 million asthmatics were found to have associated comorbidities, including allergic rhinitis, conjunctivitis, bronchiectasis, nasal congestion, and hypertensive cardiomyopathy.^6^ In Pakistan, a systematic approach to identify and manage MM in asthma, and by extension good epidemiological data reflecting this, is lacking. Meanwhile, assessing factors associated with asthma and MM in vulnerable groups aides understanding the inequities that impact disease burden and, ultimately, health outcomes.

The Obstructive Lung Disease (OLD) program^7^, integrated with primary care, has trained specialist Lung Health Nurses working with family medicine doctors to diagnose asthma using spirometry. Along with baseline characteristics, comorbidities and their types, as well as risk factors such as tobacco use and biomass fuel exposure are routinely recorded.^8^ Our study focused on determining the prevalence and patterns of MM in asthma. It also explored the relationship between the presence of MM and various demographics, including the impact of the number of co-morbidities existing at one time. This work aims to provide insights on the role of demographic determinants in influencing MM trends in asthma as a basis for strategies and best practices for prevention and targeted management in asthma.

## METHODOLOGY

An observational cross sectional retrospective study of programmatic data from 5,802 asthma patients collected between January 2021 to March 2025 was performed. The data was obtained from family medicine outpatient clinics at nine OLD integrated primary care urban and rural sites in the Indus Hospital & Health Network (IHHN).

### Ethical considerations:

Ethical approval was obtained on 1^st^ September, 2025 from the Institutional Review Board of the Indus Hospital and Health Network (IHHN_IRB_2025_09_001).

### Inclusion Criteria:

- Adults and children aged 12 years and above.
- Those with physician confirmed diagnosis of asthma.

### Exclusion Criteria:

- Patients diagnosed with an alternative chronic respiratory disease.
- Those with incomplete records.

### Data collection:

Data was captured from the Health Management Information System (HMIS) for electronic medical records of physician confirmed cases of asthma referred who underwent spirometry testing. Additional patient-reported information in confirmed cases of asthma was collected on the OLD program database in Research Electronic Data Capture (REDCap). Categorical variables included age in years, categorized as adolescents (≤19 years)^9^ and young adults (20–39 years); middle-aged adults (40–59 years) and older adults (60 years and above).^10^ Adolescents were combined with young adults due to their small sample size to enable meaningful statistical analysis. Employment status was collected (employed, homemakers, retirees, students/trainees, and unemployed individuals). Further, gender, geographical location (urban/rural), presence of MM, types and counts of comorbidities were recorded.

### Statistical analysis:

The primary outcome variable was MM in terms of presence and counts in asthma patients. Descriptive statistics assessed baseline characteristics of all asthma patients and those with MM (frequencies and percentages). Inferential analysis compared asthma patients with or without MM to find associations with age, gender, geographical location and employment status using binary and multivariate logistic regressions. Regressions were also done for establishing associations of MM counts in asthma patients with age and gender using ≤1 comorbidity as the reference. Results were presented as crude odds ratio (COR), adjusted odds ratio (AOR) and 95% Confidence Interval (CI) with p-value <0.05 considered statistically significant. For analysis, Statistical Package for Social Sciences (SPSS) version 26 was used.^11^

## RESULTS

A total of 5802 patients were diagnosed with asthma by physicians, 4177 (72%) of whom performed spirometry. Those who were unable to perform had excessive symptoms of cough and/or breathlessness. Table-I summarizes asthma patient profiles in the study.

**Table-I:** Baseline characteristics of asthma patients (n=5802).

Variables	n (%)
***Gender***	
Male	2934 (50.6)
Female	2868 (49.4)
*Age*	
Adolescents(≤19y) & Young adults (20–39y)	2037 (35.2)
Middle-aged adults (40–59y)	2308 (39.7)
Older adults (≥60y)	1457 (25.1)
***Employment status***	
Employed	2629 (45.3)
Unemployed	533 (9.2)
Homemaker	2487 (42.9)
Retired	153 (2.6)
***Geographical location***	
Rural	1675 (28.9)

### Burden and types of MM:

**Fig.1:**
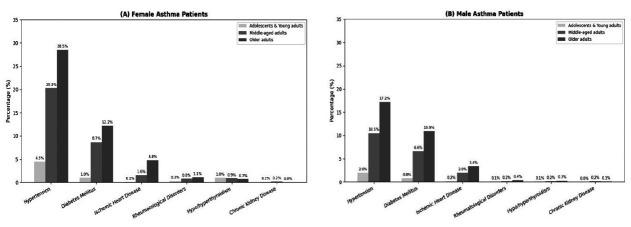
Frequencies of the top six comorbidities distributed across age groups in asthma patients, first panel representing females and second panel representing males.

Amongst asthmatics, 1102 (19%) had MM. Hypertension (13.1%) and diabetes mellitus (6.7%) were the most frequently reported comorbidities, occurring alone or in combination with other comorbidities. Ischemic heart disease (1.7%), hypo-/hyperthyroidism (0.8%), rheumatological disorders (0.5%), chronic kidney disease (0.2%), and other conditions (2.9%) were also reported. Fig.1 shows the frequencies of the top six comorbidities in asthma patients, distributed across age groups, with separate panels for females and males.

### Association of sociodemographic factors with presence of MM in asthma:

The binary and multivariate logistic regression analysis of MM in asthma patients based on the demographic factors, in terms of COR and AOR with corresponding p-values is shown in Table-II.

**Table-II:** Logistic regression of Multimorbidity in asthma patients (n= 5802) by sociodemographic determinants (COR, AOR, p-value. Reference categories for gender(female); geographical location (urban); age groups (adolescents and young adults) and employment (employed) have been indicated

Variable	Multimorbidity n=1102 Present n (%)	Multimorbidity n=4700 Absent n (%)	COR (95% CI)	p-value	AOR (95% CI)	p-value
***Gender***						
Male	481 (16.4)	2453 (83.6)	1.409 (1.23–1.60)	<0.001	1.735 (1.51–1.99)	<0.001
Female	621 (21.7)	2247 (78.3)	Reference	–	Reference	–
***Geographical location***						
Urban	832 (20.2)	3295 (79.8)	Reference	–	Reference	–
Rural	1405 (16.1)	1675 (83.9)	1.314 (1.13–1.52)	<0.001	1.296 (1.11–1.52)	<0.05
***Age groups***						
Adolescent (≤19y) & Young adults (20–39y)	127 (6.2)	1910 (93.8)	Reference	–	Reference	–
Middle-aged adults (40–59y)	520 (22.5)	1788 (77.5)	0.229 (0.18–0.28)	<0.001	0.219 (0.17–0.26)	<0.001
Older adults (≥60y)	455 (31.2)	1002 (68.8)	0.146 (0.11–0.18)	<0.001	0.131 (0.10–0.16)	<0.001
***Employment status***						
Employed	435 (16.5)	2194 (83.5)	Reference	–	Reference	–
Unemployed	14 (2.6)	519 (97.4)	0.13 (0.07–0.22)	<0.001	1.52 (1.06–2.19)	0.022
Homemaker	603 (24.2)	1884 (75.8)	1.62 (1.41–1.87)	<0.001	2.14 (1.05–4.38)	0.037
Retired	50 (32.7)	103 (67.3)	2.43 (1.71–3.45)	<0.001	1.05 (0.69–1.59)	0.812

Before running the multivariate model, multicollinearity was assessed using the Variance Inflation Factor (VIF), and all variables showed VIF values below five, indicating no significant multicollinearity among predictors.

After adjustment for all sociodemographic variables, the odds of having MM were significantly higher among middle-aged (AOR = 0.219, p < 0.001) and older adults (AOR = 0.131, p < 0.001) compared with adolescents and young adults. Male asthmatics had higher adjusted odds of MM than females (AOR = 1.735, p < 0.001). Rural residents also had increased odds of MM (AOR = 1.296, p < 0.05) compared to urban patients. Regarding employment, homemakers (AOR = 2.14, p = 0.037) and unemployed individuals (AOR = 1.52, p = 0.022) were more likely to have MM compared with employed asthmatics.

### Association between sociodemographic and number of comorbidities in asthma:

**Fig.2:**
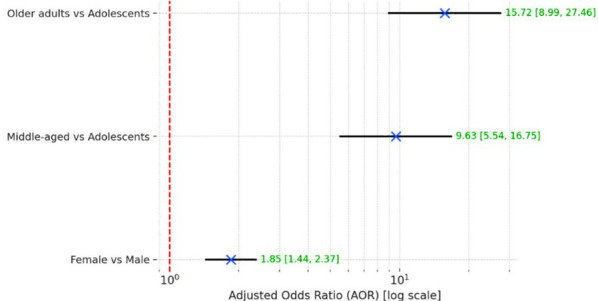
Forest plot showing adjusted odds ratios (AOR) for having more than one comorbidity among asthma patients, stratified by age group and gender (reference: ≤1 comorbidity).

Compared with adolescents and young adults, middle-aged asthmatics had 9.6 times higher odds (AOR = 9.63, 95% CI 5.54–16.75, p < 0.001) of having more than one comorbidity, after adjustment for gender. Similarly, older adults had 15.7 times higher odds (AOR = 15.72, 95% CI 8.99–27.47, p < 0.001) of having more than one comorbidity. Females were also more likely than males to have more than one comorbidity (AOR = 1.85, 95% CI 1.44–2.37, p < 0.001) (Fig.2).

## DISCUSSION

In our cohort of over 5,802 asthma patients, nearly one in five were living with at least one other chronic condition. Hypertension and diabetes mellitus were the most frequent, while ischemic heart disease, thyroid disorders, kidney disease and rheumatological disorders were less common. Although our prevalence of MM at 19% is lower than reported in international studies, it remains a clinically important finding.^12^

International data consistently shows a high burden. In Korea, over one-third of asthma patients had multiple chronic conditions, mainly cardiometabolic in nature.^12^ Results from Europe and North America are similar, linking comorbidities with worse asthma outcomes and increased healthcare utilization.^13^,^14^ In South Asia, evidence is limited, but a large regional study found cardiometabolic MM highly prevalent, particularly in urban settings and lower socioeconomic groups.^4^ Locally, a Pakistani case–control study showed cardiovascular disease in 30% of asthma patients versus 17% in non-asthmatics, reinforcing under appreciation of MM in asthma.^15^

The types of comorbidities in our study align with global patterns, where hypertension and diabetes dominate, while heart disease, thyroid disorders and arthritis are less frequent.^12^,^16^,^17^ In low- and middle-income countries, unique risks such as household biomass smoke, air pollution, and limited preventive care may contribute to this profile.^18^,^19^

Age-related patterns were notable. While older adults carried the expected burden of MM, adolescents and young adults also had unexpectedly high odds. Pakistan is a young country, with over 60% of its population under 30 years^20^,^21^, so early onset of comorbidities carries major implications for future healthcare needs. Early-life exposures including tobacco smoke, poor air quality, and recurrent infections, may predispose younger asthmatics to develop chronic conditions earlier. Survivor bias and under-diagnosis in older adults could also explain this inverse association. Nevertheless, the established relationship between advancing age and MM remains clear and is consistent with international evidence.^12^,^14^

Gender differences in among asthma patients showed an interesting pattern. Overall, more women than men had MM, yet crude and age-adjusted analyses suggested men were more likely to report at least one comorbidity. Meanwhile, women were nearly twice as likely to accumulate multiple conditions, aligning with international evidence that asthmatic females often have higher comorbidities, including obesity, arthritis, and endocrine disorders.^12^,^22^,^23^ Such differences may reflect biological vulnerability, social roles, and diagnostic practices, calling for gender-sensitive approaches to asthma care.

Socio-demographic differences were also evident. More multimorbid asthma patients were from rural areas, although rural asthmatics contributed to less than a third of our study. Given Pakistan’s largely rural population, the contributions from biomass fuel use, limited healthcare access, and environmental triggers may be significant. Homemakers, many of whom are exposed to indoor pollution, also showed higher odds, echoing evidence from rural India where women exposed to biomass smoke had higher rates of asthma and respiratory morbidity.^18^,^24^,^25^ From an economic perspective, patients not engaged in paid work were significantly more likely to have MM than those employed, highlighting the cycle of illness and socioeconomic disadvantage.

A greater number of comorbidities further contribute to poor outcomes in asthma control. These findings highlight the importance of personalized management strategies that recognize the interplay of asthma and MM, guided by treatable traits and tailored to patients’ broader social and environmental context.^22^

### Strengths:

This study has several strengths, including a large sample size from across Pakistan and physician-confirmed asthma diagnoses, enhancing the reliability of our findings. The use of standardized patient profiling allowed examination of sociodemographic determinants of MM.

### Limitations:

Some limitations must be acknowledged. Spirometry could not be performed in nearly one-third of patients, many of whom had severe symptoms, which may have introduced selection bias. The prevalence of comorbidities may also be underestimated, as data were based on patient reports during clinic visits where acute respiratory symptoms often dominate the discussion.

## CONCLUSION

This study highlights the considerable burden of MM contributing to greater complexity in asthma management, predominantly driven by hypertension and diabetes. Sociodemographic disparities were evident, with higher comorbidity odds among younger males, rural residents, and homemakers. Younger asthmatics males, rural residents, and homemakers have higher odds of at least one comorbidity whilst older adults and women are likely to be more multimorbid. This underscores the importance of MM in asthma, an under-recognized dimension of asthma care in Pakistan, requiring integrated, patient-centered approaches for screening and management. With a young demographic profile, early interventions, especially risk factor avoidance, in Pakistan could reduce the long-term burden of both asthma and MM.
